# Innate lymphoid cells control signaling circuits to regulate tissue-specific immunity

**DOI:** 10.1038/s41422-020-0323-8

**Published:** 2020-05-06

**Authors:** Christoph S. N. Klose, David Artis

**Affiliations:** 1 Department of Microbiology, Infectious Diseases and Immunology, Charité - Universitätsmedizin Berlin, 12203 Berlin, Germany; 2 Jill Roberts Institute for Research in Inflammatory Bowel Disease, Joan and Sanford I. Weill Department of Medicine, Department of Microbiology and Immunology, Weill Cornell Medicine, Cornell University, New York, NY 10021 USA

**Keywords:** Immunology, Cell biology

## Abstract

The multifaceted organization of the immune system involves not only patrolling lymphocytes that constantly monitor antigen-presenting cells in secondary lymphoid organs but also immune cells that establish permanent tissue-residency. The integration in the respective tissue and the adaption to the organ milieu enable tissue-resident cells to establish signaling circuits with parenchymal cells to coordinate immune responses and maintain tissue homeostasis. Innate lymphoid cells (ILCs) are tissue-resident innate immune cells that have a similar functional diversity to T cells including lineage-specifying transcription factors that drive certain effector programs. Since their formal discovery 10 years ago, it has become clear that ILCs are present in almost every tissue but strongly enriched at barrier surfaces, where they regulate immunity to infection, chronic inflammation, and tissue maintenance. In this context, recent research has identified ILCs as key in orchestrating tissue homeostasis through their ability to sustain bidirectional interactions with epithelial cells, neurons, stromal cells, adipocytes, and many other tissue-resident cells. In this review, we provide a comprehensive discussion of recent studies that define the development and heterogeneity of ILC populations and their impact on innate and adaptive immunity. Further, we discuss emerging research on the influence of the nervous system, circadian rhythm, and developmental plasticity on ILC function. Uncovering the signaling circuits that control development and function of ILCs will provide an integrated view on how immune responses in tissues are synchronized with functional relevance far beyond the classical view of the role of the immune system in discrimination between self/non-self and host defense.

## Introduction

The transition of the innate to the adaptive immune response takes place in the secondary lymphoid organs where antigen-presenting cells present peptides via major histocompatibility complex (MHC) molecules to patrolling naïve T cells, which scan numerous cells searching for their specific antigen. While activation of the adaptive immune response in the lymphoid structure is key in fighting infections and forming immunological memory, it also became apparent that diverse immune cells including innate and adaptive lymphocytes establish permanent tissue-residency in peripheral organs thus creating a first-line defense system against invading pathogens at barrier surfaces.^1^ Among tissue-resident cells, innate lymphoid cells (ILCs) emerge as populations of innate immune cells that have lymphoid morphology but lack rearranged antigen receptors.^2^ As tissue-resident cells, ILCs adapt to the organ milieu and establish close interactions with hematopoietic cells but also with non-hematopoietic cells in the tissues including neurons, epithelial cells, stromal cells, and other parenchymal cells, such as adipocytes and hepatocytes.^3,4^ The challenge in the upcoming years will be to uncover the cellular and molecular basis of these tissue-resident circuits, which regulate inflammation and tissue homeostasis in health and disease.

ILCs are classified into different groups depending on their developmental and effector program defined by the expression of lineage-specifying transcription factors (TFs).^2,5^ The expression of a lineage-specifying TF is selective to a certain degree, sometimes not exclusive though, as in the case of GATA binding protein 3 (GATA-3) or T-box expressed in T cells (T-bet), which are expressed in several ILC lineages. Further, the lineage-specifying factor is essential for the development of the corresponding ILC lineage. Similar to T cells and based on developmental, functional, and migratory aspects, mature ILCs can be divided into cytotoxic (interleukin-7 receptor α^−^, IL-7Rα^–^) and non-cytotoxic (or helper-like, IL-7Rα^+^) ILCs.^2,5^ Natural killer (NK) cells are the oldest member of the ILC family and the only representative of cytotoxic ILCs. NK cells express the lineage-specifying T-box TF Eomesodermin (Eomes), and mediate effector functions via perforin-dependent cytotoxicity and production of IFN-γ, which together protect from intracellular pathogens and tumors.^6,7^ Non-cytotoxic ILCs are divided into three major groups coined ILC1, ILC2, and ILC3. Similar to NK cells, ILC1s promote type 1 immune responses against intracellular pathogens via the production of IFN-γ and TNF. However, ILC1s lack perforin-dependent cytotoxicity but they are able to kill target cells via alternative pathways, such as TNF receptor-mediated induction of apoptosis. ILC1s do not develop in the absence of the TF T-bet, which is evolutionarily related to Eomes.^6,8^ ILC2s depend on the lineage-specifying TF GATA-3 and secrete classical type 2 effector cytokines, such as interleukin (IL)-4, IL-5, IL-9, and IL-13, as well as amphiregulin (AREG), which promote type 2 inflammation in the context of anti-helminth immunity, allergic reactions or tissue remodeling.^9–14^ ILC3s comprise of different populations of retinoic acid receptor-related orphan receptor (ROR)γt-dependent lymphocytes, which secrete IL-22 to fortify epithelial barriers.^15–17^ These include fetal lymphoid-tissue inducer (LTi) cells,^18^ which are essential for the formation of secondary lymphoid tissues during development. In adult mice, CCR6^+^ ILC3s have a similar phenotype to LTi cells and are therefore referred to as LTi-like lymphocytes. In the intestine, they are located in cryptopatches and produce IL-17 in addition to IL-22.^19–21^ The second subset of ILC3s in adult mice lacks CCR6 expression (CCR6^−^ ILC3s) but co-expresses the TF T-bet in addition to RORγt. Upregulation of T-bet is establishing a transcriptional program in these cells, which is similar to one of ILC1s (ILC1-like) characterized by loss of the lineage-specifying TF RORγt and upregulation of natural killer-cell receptors and markers of type 1 immunity, such as NKp46, NKG2D, NK1.1, and IFN-γ.^21–25^

NK cells have a similar migratory pattern to T and B cells and express the adhesion molecule CD62L, which allows them to transmigrate through high endothelial venules (HEVs) from the bloodstream into lymphoid structures.^6^ As a consequence, NK cells are well represented in secondary lymphoid organs, whereas other ILC subsets, which lack CD62L are predominantly tissue-resident lymphocytes.^26^ As tissue-resident cells, ILCs integrate into the organ milieu and only circulate in the bloodstream in low numbers but are often replenished by local precursors.^27–29^ This notion is supported by parabiosis experiments where mainly donor-derived adaptive lymphocytes and NK cells, but not other ILCs subsets could be recovered from the recipient’s tissues at steady state.^26,30,31^ During worm infection, ILC2s and in particular inflammatory ILC2s can leave their environment and are disseminated to other tissues via the bloodstream.^32–34^ Because of their localization at barrier surfaces, which are often exposed to pathogens, allergen or irritants, ILCs are first responders during immune activation and thereby influence the cytokine milieu and the adaptive immune response. Uncovering how ILCs are regulated and how they interact with tissue-resident cells to maintain organ homeostasis is key to understand how protective or detrimental immune responses in tissues are generated.

## ILC development

**Fig. 1:**
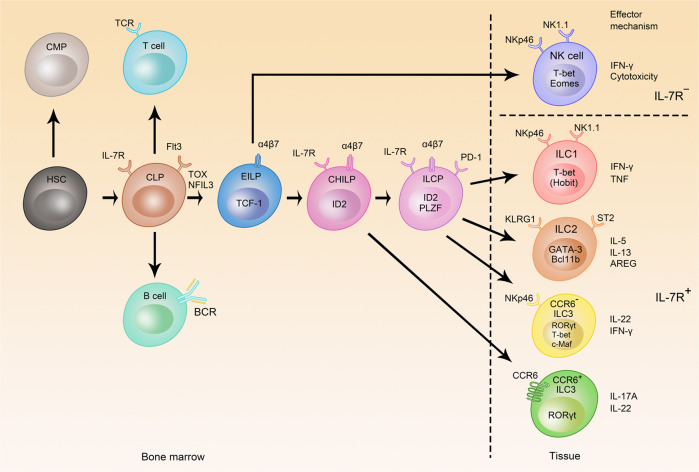
Development of ILCs. The hematopoietic stem cell (HSC) is the source of all hematopoietic cells and give rise to the common myeloid progenitor (CMP) and common lymphoid progenitor (CLP), and CLP has the potential to develop into all lymphocytes. The early ILC progenitor (EILP) gives rise to all ILC subsets, whereas common helper-like ILC progenitor (CHILP) and the ILC progenitor (ILCP) still have multi-lineage potential but this is restricted to ILC1, ILC2, and ILC3 as indicated by arrows. Transcriptional requirements, phenotypical markers, and effector molecules of ILCs are shown (TCR T cell receptor, BCR B cell receptor).

In the bone marrow, hematopoietic stem cells are the origin of the two major hematopoietic branches, the myeloid and the lymphoid lineage represented by the common myeloid precursor (CMP) and common lymphoid precursor (CLP), respectively.^35,36^ The CLP is phenotypically defined as CD127^+^ (IL-7Rα^+^) Flt3^+^ c-Kit^int^ Sca-1^int^ cells among lineage-negative (Lin^−^) cells, illustrating the importance of IL-7 for the development of the lymphoid lineage, and is giving rise to both innate and adaptive lymphocytes.^37^ Innate and adaptive lymphocyte development diverges after the CLP stage and committed precursor cells could be defined, which still possess a multi-lineage potential for different ILC lineages, but not for adaptive lymphocytes^6,38,39^ (Fig. 1). The earliest stage of these committed innate precursors, coined early ILC precursor (EILP), is defined by expression of the TF T cell factor 1 (TCF-1) and has the potential to give rise to all ILC subsets.^38^ Somehow counterintuitive is the low expression of IL-7Rα on the EILP because upstream (CLP) and downstream precursors have high IL-7Rα expression. Nevertheless, it was demonstrated that EILP was upstream of subsequent precursors and did not constitute an alternative route of ILC development.^40^ The modulation of IL-7Rα during development is not unprecedented during lymphocyte development since double-negative thymocytes have high IL-7Rα expression until it is downregulated at the double-positive stage and then again upregulated in single-positive thymocytes.^41^ The common helper-like ILC progenitor (CHILP) is defined by a surface phenotype similar to CLP, including IL-7Rα expression but lack of CD93 and Flt3, and expression of the transcriptional repressor ID2.^6^ The analysis of the *Id2*-deficient mice revealed that these mice lacked all ILC subsets and had a maturation defect in NK cells.^11,42,43^ While the CHILP had multi-lineage potential documented in several publications, more heterogeneity was found within the population, e.g., the TF PLZF using multicolor approaches.^39^ Reporter mice and fate-labeling approaches revealed that although ILCs lacked expression of PLZF, they had a history of PLZF expression suggesting that they were derived from a PLZF^+^ progenitor cell.^44^ The PLZF^+^ progenitor cell had a similar phenotype to the CHILP but lacked the potential to give rise to CCR6^+^ ILC3s. Several approaches including single-cell sequencing confirmed the existence and potential of multi-lineage ILC precursors and revealed additional markers,  such as PD-1, which is highly expressed on PLZF^+^ ILC precursors (ILCP).^45,46^ Key TFs for early ILC fate commitment in addition to TCF-1, ID2, and PLZF include NFIL3, TOX, and GATA-3.^47–51^ In line with their role in early ILC development, gene-deficient mice for one of these TFs exhibit a strong developmental defect in all or most ILC lineages except NK cells and LTi cells in GATA-3-deficient mice. For a more detailed discussion about the differentiation potential and transcriptional regulation of ILC precursors, the reader is referred to recently published reviews that extensively discuss this topic.^5,52^

A plethora of TFs was found to either affect development or maturation of NK cells including ETS-1, PU.1, IKAROS, EOMES, ZEB2, AIOLOS, PRDM1 (BLIMP1), FOXO1, IRF2, RUNX3, and KLF2.^52^ Among them, Eomes appears as relatively selective because it is not expressed in other ILC subsets, but in CD8^+^ T cells in mice; and conditional gene targeting of *Eomes* using *NKp46*Cre resulted in the depletion of NK cells without affecting other innate or adaptive lymphocytes.^7,53,54^ However, Eomes expression was found in human ILC1s in some tissues and, therefore, is not a selective marker to distinguish NK cells and ILC1s in humans.^55^ It should be noted that in contrast to ILC1s, NK cells were only mildly affected by the lack of T-bet resulting in altered migration, but unperturbed development of NK cells.^56^ In contrast, ILC1 development is strictly dependent on T-bet but not on Eomes.^6,8,57^ The TF Hobit was discovered as an essential regulator for the development of tissue-resident T cells,^58^ and was later shown to be essential for the development of liver ILC1s but not ILC1s in other organs.^59^

The development of ILC2s depends on the TF GATA-3, BCL11b, RORα, and Gfi1.^9,14,60–62^ GATA-3 is considered as lineage-specifying TF although it is required for the development of multiple ILC lineages because GATA-3 is an essential regulator of early ILC development on the CHILP level. Hence, GATA-3 is similarly active as in T cells, in which GATA-3 regulates not only T helper (Th)2 fate decision but also several steps during development including early lineage commitment.^47,63^

The development of all ILC3 subsets strictly depends on the lineage-specifying TF RORγt.^19,64,65^ CCR6^+^ LTi-like ILC3s are developmentally affected in TOX-, NFIL3-, or GATA-3-deficient mice,^48,51,66^ and these three TFs mediate their function in early ILC development. While CCR6^+^ ILC3s developed in mice deficient in the aryl hydrocarbon receptor (AHR), they were functionally impaired, illustrated by the altered maturation of cryptopatches in *Ahr*^−/−^ mice, whereas postnatally emerging CCR6^−^ ILC3s did not develop.^21,67,68^ CCR6^−^ ILC3s develop after birth and co-express T-bet, which is competing with RORγt in determining the cell fate of ILC3s^21–23^ and which is establishing a type 1 program accompanied by a loss of RORγt and IL-22 production and upregulation of NK receptors and T-bet target genes, such as NKp46, NKG2D, and NK1.1, IL12Rβ2, CXCR3, and IFN-γ.^24,25^ In the absence of T-bet, CCR6^–^ ILC3s still developed but failed to induce the type 1 program. Notably, T-bet-deficient ILC3s could still trigger colitis through secretion of IL-17A.^21,69^ The balance between RORγt and T-bet is regulated by the TF c-Maf, which repressed T-bet expression by binding to the promoter and therefore suppressing the type 1 program in ILC3s.^70,71^

## NK cells

### Immune recognition strategies used by NK cells

**Fig. 2:**
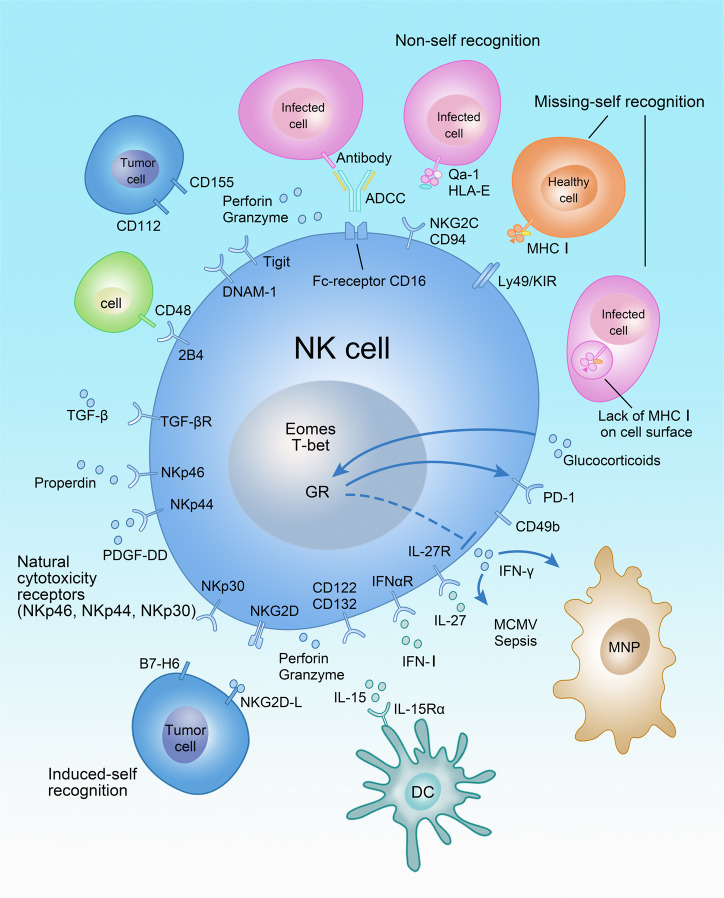
Regulation of NK-cell activation. NK cells are regulated by recognition of non-self, missing-self, and induced-self ligands. Receptor-ligand interactions and factors regulating NK-cell activation as well as effector functions are shown. MNP mononuclear phagocyte, DC dendritic cell, GR glucocorticoid receptor (Nr3c1), ADCC antibody-dependent cellular cytotoxicity.

Immune recognition in both the innate and adaptive immune system relies to a large extent on the interaction between immunoreceptors and the corresponding ligands. Moreover, such immunoreceptors in most cases detect non-self peptides with respect to the T cell receptor (TCR) or B cell receptor (BCR), or ligands recognizing a broad biochemical spectrum in case of pattern recognition receptors.^72^ While NK-cell-mediated immune surveillance is similarly dominated by receptor-ligand interaction, immune recognition strategies used by NK cells exceed non-self recognition and are mainly based on recognition of self-molecules, coined missing-self and induced-self recognition (Fig. 2).^73^ Notably, the immune regulation of NK cells by missing-self and induced-self ligands is very dominant resulting in altered NK development when missing-self and induced-self ligands are absent.^74,75^ Immune recognition strategies of NK cells have similarities to T cells, because non-self, missing-self, and induced-self either recognizes classical MHC I (missing-self) or non-classical MHC I molecules in case of some non-self and induced-self ligands. Missing-self recognition is based on inhibitory receptors of the Ly49 (mouse) or killer-cell immunoglobulin-like receptor (KIR, human) family expressed on NK cells, which detect classical MHC I on target cells resulting in their lysis in the absence of MHC I.^76^ Concerning non-self recognition, non-classical MHC I molecules Qa-1/HLA-E present foreign peptides derived from murine cytomegalovirus (MCMV) to the NKG2C/CD94 receptor expressed on NK cells thus forming a complementary innate receptor-ligand pair to the peptide MHC-TCR interaction in the adaptive immune system.^77^ In contrast, ligands for the stimulatory immunoreceptor NKG2D are non-classical MHC I molecules that do not present peptides and that are not expressed by healthy cells, but that are upregulated upon infection or transformation in tumor cells.^78^ Likewise, B7-H6, which has structural similarities to the B7 co-stimulatory molecules, was induced on tumor cells and stimulated anti-tumor immunity via binding to NKp30.^79^ NKp30 belongs together with NKp44 and NKp46 to the family of natural cytotoxicity receptors.^80^ NKp44 recognized PDGF-DD^81^ and thereby acted as a factor, which promoted tumor growth, whereas NKp46 bound complement factor P (properdin) and was involved in the control of *Neisseria meningitides* infections.^82^ Likewise, NK cells recognize the fragment crystallizable (Fc) portion of antibody via the Fc receptor CD16 and lysed antibody-coated cells by antibody-dependent cellular cytotoxicity (ADCC). NK cells integrate stimulatory or inhibitory signals from self-ligands, including but not limited to Tigit, DNAM-1, 2B4, and PD-1, which define the activation threshold or cell adhesion of NK cells.^5,52^

In addition to membrane-bound receptor-ligand interaction, NK cells are regulated by humoral factors e.g. cytokines, such as IL-15, IFN-I, IL-27, IL-12, and TGF-β, but also glucocorticoids. IL-15 is essential for the development and activation of NK cells and is often trans-presented via the IL-15Rα-chain expressed by dendritic cells (DCs) to the low-affinity IL-2/IL-15 receptor on NK cells composed of the IL-2Rβ-chain CD122 and the common γ-chain CD132.^83^ DCs produce additional cytokines,  such as IFN-I, IL-27, and IL-12 that are required for priming and activation of NK cells.^84^ While IL-12 was originally described as an NK-cell-stimulating factor,^85^ several publications reported its stronger effects on ILC1s or ILC3s than on NK cells.^6,59,84,86^ Moreover, NK cells were responsive to glucocorticoid signals via expression of the nuclear receptor Nr3c1 (glucocorticoid receptor) and were therefore regulated by neuroendocrine signals from the hypothalamic-pituitary-adrenal axis. Glucocorticoids prevent IFN-γ production by NK cells in conjunction with the inhibitory receptor PD-1 and thus control susceptibility to MCMV infection and sepsis.^87,88^

In summary, NK cells are patrolling innate lymphocytes that test target cells for the presence and absence of ligands to eliminate the target cell if necessary. Additional cytokine signals, such as IL-15, IFN-I, and IL-27 regulate NK-cell development and activation.

### NK cells fight intracellular infections and tumors via cell-mediated cytotoxicity and production of IFN-γ

NK-cell activation is to a large extent regulated by the balance between stimulatory and inhibitory signals received by their receptors. If the activation threshold is exceeded, a response is triggered, which results in the specific lysis of the target cell or secretion of the cytokine IFN-γ. To mediate cytotoxic activity, the cytoskeleton is reorganized toward the target cell, and an immunological synapse is formed resulting in the release of granules that contain perforin and granzymes. Perforin is a pore-forming molecule, which ruptures the plasma membrane of the target cell and granzymes are proteases that induce apoptosis via different mechanisms including cleavage of caspase 3. The target cell is often an infected cell, which is removed via cell-mediated cytotoxicity to control the infection. Elimination of hematopoietic cells via cell-mediated cytotoxicity was described as an immune regulatory mechanism as well, e.g., during infection with lymphocytic choriomeningitis virus (LCMV).^89,90^ IFN-γ is an equally important effector molecule produced by NK cells since it activates antimicrobial functions in macrophages, increases antigen presentation and immunoglobulin (Ig) class switching. Deficiency in either perforin or IFN-γ production results in susceptibility to infection with MCMV, a viral infection that is largely controlled by NK cells (Table 1).^91–93^ MCMV belongs to the β-herpesvirus family of double-stranded DNA viruses that establish long term persistent infection in the host by manipulating the immune response and in particular MHC I expression and recognition by NK cells via NKG2D ligands. MCMV encodes several proteins that interfere with antigen presentation, of which m157 mimics MHC I, probably to avoid missing-self recognition by NK cells through the engagement of inhibitory Ly49 receptors. Some mouse strains have developed a stimulatory Ly49 receptor called Ly49H to prevent immune evasion by MCMV. Ly49H recognized m157 and dominated the immune responses to MCMV illustrated by superior viral control in mouse strains that carry Ly49H.^94^

**Table 1:** Disease association of ILC subsets.

	NK cells	ILC1	ILC2	ILC3
Protective immunity against pathogens	*Cytomegalovirus*^91,94^ *Toxoplasma gondii*^235^	*Clostridium difficile*^113^ *Toxoplasma gondii*^6,235^ *Cytomegalovirus*^59^	Helminth parasites^10–12,150,151,154^	*Citrobacter rodentium*^16,187,189,190^ *Clostridium difficile*^113^ *Candida albicans*^196–198^ *Rotavirus*^178^ *Yersinia enterocolitica*^200^ *Mycobacterium tuberculosis*^196,197^ *Salmonella Typhimurium*^186^ *Listeria monocytogenes*^175^
Allergy, autoimmunity, overshooting immune responses	Contact hypersensitivity^230^ Sepsis^87^	Contact hypersensitivity^30^ Anti-CD40-induced colitis^55^ EAE^53^ Sepsis^87^	Allergic Asthma/airway hyperreactivity^157,158^ Atopic dermatitis^140,156,161^ Chronic rhinosinusitis^13^ Hepatitis^165^	Colitis, intestinal inflammation or immunopathology^17,21,25,69,177,189,190,201^ HFD-induced asthma^199^ Psoriasis^239^
Tissue repair, remodeling, integrity, and homeostasis	Uterus/placenta^104^	Liver injury^114^ Metabolic homeostasis^103^	Resolution of arthritis^143^ Fibrosis^167^ Wound healing^147,163,164,166^ Metabolic homeostasis and insulin resistance^245,246,248^	Regeneration of intestinal epithelium or thymus^192–194^ Barrier integrity^15,184^ Fucosylation of intestinal epithelium^186^ Metabolic homeostasis^224,249^
Cancer	Anti-tumor immunity^96,97,99^	TRAIL-mediated anti-tumor immunity^107^	– (Suggested by some studies)	Resistance to IL-12-secreting melanoma^86^ Colitis-associated cancer^192,195^
Organ development	–	–	–	Lymph node organogenesis^18,19,42^

ILCs were linked to numerous physiological processes in mice and humans, sometimes underlying the pathogenesis of diseases as listed here. Numbers denote the reference number of the corresponding publications. EAE experimental autoimmune encephalomyelitis; HFD high-fat diet.

The immunosurveillance of tumors by NK cells is controlled by immunoreceptors, such as NKG2D, NKp30, and NKp44. We will focus here on anti-tumor immunity mediated by NKG2D because it is best understood due to the availability of knockout mouse models. Ligands for the NKG2D receptor were found to be expressed on stressed or transformed cells, but not on healthy cells.^95^ Ectopic overexpression of NKG2D ligands on tumor cells resulted in the rejection of the tumor cell line by the immune system and even formation of immunological memory.^96^ Further, NKG2D-deficient mice were more susceptible to epithelial and lymphoid tumors that expressed NKG2D ligands.^97^ While shedding of NKG2D ligands on the surface of tumor cells was interpreted as a tumor escape mechanism,^98^ recent data suggest that shedding might prevent the downregulation of NKG2D and therefore promote anti-tumor immunity.^99^ Clinical trials are undertaken to investigate how this basic knowledge about checkpoints of NK-cell regulation could be translated to trigger anti-tumor immunity in patients.^100^

## ILC1s

The term ILC1 includes several populations of innate lymphocytes that are developmentally distinct from conventional NK cells. ILC1s were described in many tissues including TNF-related apoptosis ligand (TRAIL)^+^ NK cells/ILC1s in the liver,^101^ thymic NK cells,^102^ ILC1s in the intestinal lamina propria^6^ and epithelium,^55^ in adipose tissue^103^ and in the uterus.^104^ In most tissues and with the exception of thymic NK cells,^102^ ILC1s developmentally depend on the TF T-bet and the cytokine IL-15 but not on IL-7, although they express IL-7R.^6,8^ Since ILC1 populations found in different tissues sometimes have a slightly different phenotype or developmental requirements, it is still unclear whether this reflects tissue adaption within one lineage or several lineages of innate lymphocytes. Further work is needed to elucidate these aspects.

**Fig. 3:**
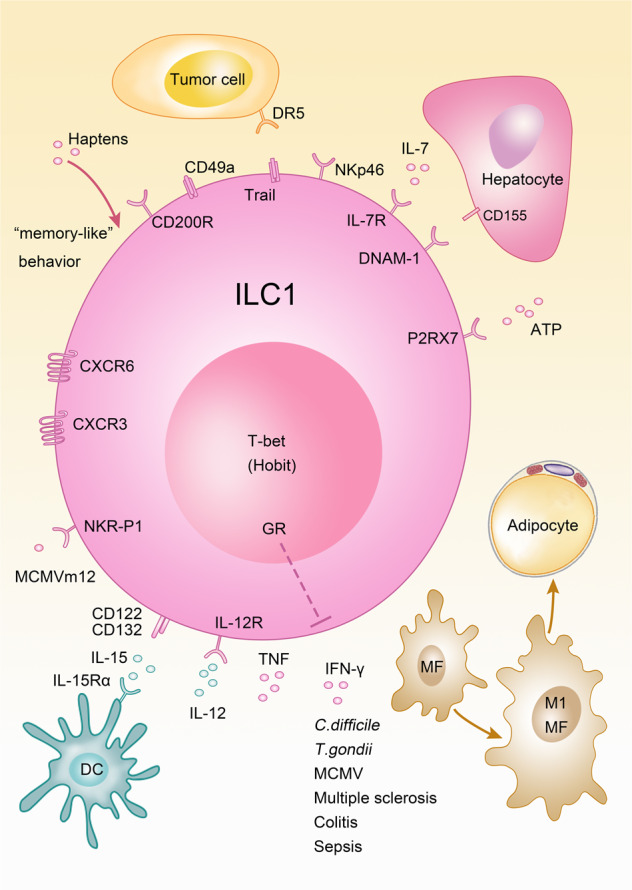
Immunoregulation mediated by ILC1s. Multiple interactions between ILC1 and hematopoietic or parenchymal cells are depicted. MF macrophage, DC dendritic cell, MCMV murine cytomegalovirus, GR glucocorticoid receptor (Nr3c1).

Although ILC1s expressed a similar array of killer receptors as NK cells, such as NKp46, NKG2D, and NK1.1, the functional implications of these receptors for ILC1 biology remain understudied (Fig. 3). However, a point mutation in NKp46 was recently identified in CD45.1 mice, which prevented membrane trafficking of the receptor. As a consequence, ILC1s from mice carrying the point mutation in NKp46 had diminished surface expression of TRAIL resulting in decreased anti-tumor immunity.^105,106^ Moreover, this mutation also affected the function of NK cells and NKp46^+^ ILC3s, which all express NKp46.^105,106^

Ly49 receptors are strongly underrepresented on ILC1s suggesting that ILC1s do not rely on missing-self recognition.^6,8^ ILC1s were first described in the liver as TRAIL^+^ NK cells and based on their phenotype and different functional properties they were interpreted as immature NK cells.^101^ Although TRAIL could be induced on activated classical NK cells, it is highly expressed at steady state on ILC1s. TRAIL can engage DR4/DR5 receptors on the target cell and induce apoptosis through Fas-associated protein with death domain (FADD) as well as caspase-8 and -10, a pathway different from perforin/granzyme-mediated cytotoxicity of NK cells. While this pathway has been linked to anti-tumor immunity, the resistance of cancer cells to TRAIL-mediated apoptosis has also been reported.^107^

A very intriguing aspect of ILC1 biology is their memory-like property. Although poorly understood on a molecular level, the concept is based on the finding that NKp46^+^ lymphocytes from the liver (ILC1) but not from the spleen could trigger hapten-specific immune responses leading to skin contact hypersensitivity, which did not require the adaptive immune system.^108^ ILC1 memory was formed following hapten sensitization of the skin in the skin draining lymph nodes and required IL-7 and CXCR3 signaling. Moreover, the homing of ILC1s to the liver was mediated by CXCR6 on ILC1s and the ligand CXCL16.^109,110^ The ILC1 memory response was not limited to contact hypersensitivity but was also shown to mount antigen-specific recall responses, which mediated immunity against viruses including vesicular stomatitis virus, influenza and human immunodeficiency virus type 1 (HIV-1).^110^ While antigen-specific memory responses were linked to ILC1s,^108,109^ ILC2s were shown to adopt a memory-like phenotype and function after papain challenge or IL-33 exposure in an antigen-unspecific manner.^111^

Although originally believed to be limited to adaptive lymphocytes, memory-like behavior of ILCs was also reported. However, unlike the molecular machinery regulating adaptive memory responses, the receptors mediating antigen-specific memory of ILC1s require further investigation.

In addition to memory responses, ILC1s can mediate protection against acute infections, such as MCMV infection. *Hobit*^−/−^ mice, which lack liver ILC1s, were more susceptible to MCMV infection in the liver at early time points. This is a remarkable finding because MCMV is controlled at later stages by NK cell via Ly49H receptors.^94^ Mechanistically, ILC1s were stimulated by IL-12 derived from conventional dendritic cells to secrete IFN-γ^59^ and did also mount a memory response driven by the MCMV protein m12.^112^ ILC1s contributed to host defense against infection by secretion of IFN-γ and TNF in mouse models of *Clostridium difficile* and *Toxoplasma gondii* infection, but were also implicated in the development of chronic or excessive inflammation during colitis, multiple sclerosis, and sepsis.^6,8,53,55,87,113^ The effect of ILC1-derived IFN-γ following IL-12 release from myeloid cells was not limited to infection, but also regulated macrophage polarization towards an M1 phenotype, which promotes obesity and insulin resistance.^103^

Protective effects of ILC1s were described during sterile inflammation in the liver after injection of carbon tetrachloride which triggered acute liver injury. ILC1s were activated via DNAM-1 and the ligand CD155 expressed on hepatocytes together with the release of IL-7 and ATP, which stimulate IFN-γ production from ILC1s. IFN-γ induced survival signals in hepatocytes mediated by the anti-apoptotic protein Bcl-XL.^114^

Although development and regulation of NK cells and ILC1s are distinct, the overlap in effector functions, such as IFN-γ and promotion of type 1 immunity together with the limitations in specifically targeting these subsets has thus far hindered dissection of the unique functions of ILC1s, and further research will be required to address this question.

## ILC2s

### Regulation of ILC2s

Immune recognition strategies of the innate immune cells, such as myeloid cells are largely dominated by pattern recognition receptors for a big variety of non-self molecules and presentation of non-self peptides to adaptive lymphocytes via MHC. Upon pathogenic encounter, myeloid cells secrete cytokines and migrate to secondary lymphoid organs to present non-self peptides to adaptive lymphocytes.^72^ This provokes the question of whether ILCs have similar properties to their innate myeloid counterpart in terms of sensing via pattern recognition receptors and antigen presentation.

While reports about pattern recognition receptors on helper-like ILCs are scarce, presentation of peptide-MHC was found to regulate adaptive immune response as discussed in section ‘Regulation of adaptive immune responses by ILCs’ in this review. Although membrane-bound receptor-ligand interactions regulate helper-like ILC functions, immune receptor-ligand interaction appears less dominant in regulating these cell subsets compared with soluble factors. This is also in line with the concept of tissue-resident cells, which are embedded into the fabric of tissues and require diffusible effectors as opposed to patrolling immune cells.

**Fig. 4:**
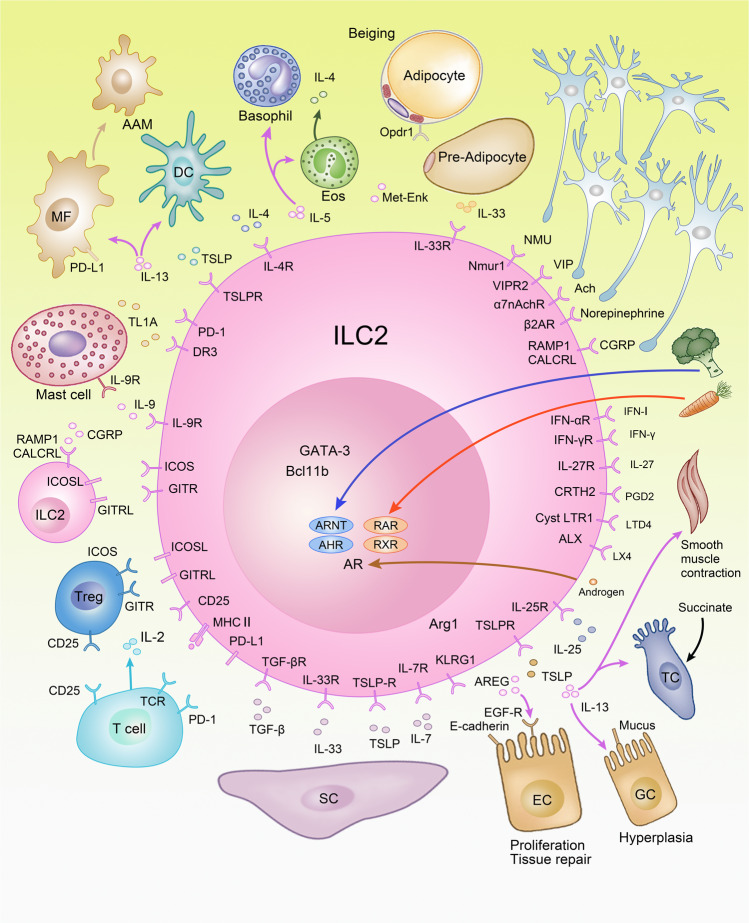
ILC2s regulate type 2 inflammation in tissues. ILC2s maintain interactions between neurons, epithelial cells, stromal cells, adipocytes but also myeloid cells and adaptive lymphocytes to regulate tissue homeostasis. Baso basophil granulocyte, Eos eosinophil granulocyte, MF macrophage, DC dendritic cell, SC stromal cell, AAM alternatively activated macrophage, AR androgen receptor, AHR aryl hydrocarbon receptor, Arnt nuclear translocator of AHR, RAR retinoic acid receptor, RXR retinoid X receptor, Arg1 arginase, IgA immunoglobulin A, EC epithelial cell, GC goblet cell, TC tuft cell.

ILC2s are mainly regulated by soluble factors including cytokines, neuronal factors, inflammatory mediators, and hormones. In addition to IL-2 and IL-7, which are broadly sensed by ILCs and adaptive lymphocytes, the cytokines IL-4, TNF-like ligand 1 A (TL1A), transforming growth factor (TGF)-β, stem cell factor (SCF) stimulate ILC2 activation and the alarmins IL-25, IL-33, and thymic stromal lymphopoietin (TSLP) are major activators of ILC2s (Fig. 4).^10–12,115–117^ IL-25 is secreted by specialized epithelial cells called tuft cells, which express chemosensory receptors detecting succinate production by worms and protozoans.^118–122^ IL-25 is released following helminth infections and triggers activation and generation of a subset of ILC2s coined inflammatory ILC2s. Inflammatory ILC2s promote anti-helminth immunity and were described in the mesenteric lymph nodes and lungs following worm infection but were barely detectable at steady state in these organs.^32,33^ However, intestinal ILC2s, which express high amounts of the IL-25-receptor component IL-17RB and low amounts of the IL-33-receptor subunit ST2, are phenotypically similar to inflammatory ILC2s. Therefore, these cells were proposed to differentiate into inflammatory ILC2s and migrate to the mesenteric lymph nodes upon worm infection.^4,32,33,120,123^ Generation of inflammatory ILC2s was promoted by the enzyme tryptophan hydroxylase 1, which was required for anti-helminth immunity and which was induced in ILC2s following worm infection or IL-33 treatment.^123^ In contrast to IL-25, recent data revealed that IL-33 was expressed in PDGFRα^+^ stromal cell and pre-adipocytes in different tissues including the adipose tissue, lung and intestine.^123–127^ Finally, adventitial stromal cells express IL-33 and are a major source of TSLP. These data indicate that non-hematopoietic cells,  such as Tuft cells or adventitial stromal cells might be signaling hubs in tissues that are specialized in triggering type 2 immune responses. ILC2 activation is further regulated by inflammatory mediators of the arachnoid acid pathway, such as prostaglandin (PG) D2 and leukotriene D4 via CRTH2 and CysLTR1, respectively. In contrast, PGE2 and lipoxin A4 (LX4) inhibited ILC2 activation via EP4 and ALX receptors, respectively.^128–131^ Additional negative regulators of ILC2 function include cytokines that promote type 1 immune responses,^31,132,133^ such as type I and II interferons, as well as IL-27 but also glucocorticoids,^87,88^ male sex hormones^134^ and some metabolites. Interestingly, the dietary metabolite retinoic acid contained in carrots and the phytochemical indole-3-carbinol, which is an AHR ligand found in cruciferous vegetables, such as cabbage and broccoli, restrain ILC2 responses, whereas they stimulate ILC3s. Consequently, dietary metabolites can shape the immune responses via regulation of ILC2s and ILC3s as demonstrated during helminth and *Citrobacter rodentium* (*C. rodentium*) infections.^135–138^

ILC2s are phenotypically defined by expression of the inhibitory receptor KLRG1, which recognizes E- and N-cadherins, and which could therefore transduce inhibitory signals upon ligand binding. However, although inhibitory effects of KLRG1 on human ILC2s were reported in vitro, the in vivo relevance remains to be demonstrated. In this context, it should be noted that the genetic ablation of *Klrg1* did not alter T cell activation in vivo, nor did it lead to hyperreactive T cells.^139,140^ Further, ILC2s provide co-stimulatory signals to each other using inducible T cell co-stimulator ligand (ICOSL) and glucocorticoid-induced TNFR-related ligand (GITRL) combined with autocrine or paracrine secretion of IL-9.^133,141–144^ ILC2s are repressed via PD-1 that is expressed on activated ILC2s, and blocking or genetic deletion of PD-1 results in exaggerated type 2 inflammation promoting allergic asthma or worm resistance.^45,145^ ILC2s maintain multiple interactions in tissues with both hematopoietic and non-hematopoietic cells to regulate type 2 inflammation (Fig. 4). Among the strongest stimulators of ILC2s are alarmins, which are secreted by specialized epithelial cells and stromal cells.

### Effector functions of ILC2s

ILC2s mediate their function mainly by secretion of soluble effector molecules, in particular classical type 2 cytokines IL-5, IL-9, and IL-13 and under some conditions IL-4.^10–12,144,146^ In general, these cytokines promote type 2 inflammation by acting on diverse cell types. IL-9 is mainly produced by ILC2s in an autocrine or paracrine manner thus leading to the expansion of ILC2s, which also carry the IL-9 receptor. IL-9-dependent expansion of ILC2s was shown to be crucial for tissue repair following lung inflammation or resolution of arthritis.^143,144,147^ IL-5 is important for activating innate myeloid cells, which are involved in type 2 immunity. In particular, IL-5 mediates recruitment and activation of eosinophils, which secrete IL-4, further promoting type 2 inflammation during allergic airway inflammation but also metabolic homeostasis.^148^ While IL-13 promotes type 2 inflammation via differentiation of alternatively activated macrophages (AAMs), it is the direct effector cytokine promoting worm expulsion via stimulating smooth muscle contraction and hyperplasia of goblet cells resulting in enhanced mucus production as well as IL-25 secretion from tuft cells to further boost type 2 inflammation.^3^ IL-25 is a strong inducer of ILC2 activation and inflammatory ILC2s. Such stimuli promote migration of ILC2s from their tissue of origin to the blood and dissemination to other tissues where they promote systemic type 2 inflammation.^32–34^

It is well established that type 2 immunity promotes expulsion of helminth parasites and noxious environmental substances as well as tissue repair.^149^ Since the crucial cytokine IL-13 is secreted by both Th2 cells and ILC2s, it raises the question about the contribution of both cell types to the immune response against  helminths. Published evidence suggests a pivotal role of ILC2s in immunity against *Nippostrongylus brasiliensis (N. brasiliensis)*,^10,11^ *Strongyloides venezuelensis*,^150^ and *Trichuris muris*^151^ infections but we will focus the discussion on *N. brasiliensis* since most data are available from this infection model. While *Rag*^−/−^ mice have comparable worm counts as wild-type mice at early time points following *N. brasiliensis* infection, worm expulsion is delayed at day 10 post infection. These data suggest that T cells are dispensable during the early infection phase but mediate worm expulsion at later time points or during reinfection.^10,152^ It was also demonstrated that injection of recombinant IL-25 or IL-33 promoted worm expulsion in the absence of T cells.^10,12,153^ Furthermore, Oliphant et al. depleted ILC2s without affecting T cell numbers and reported a higher worm burden in ILC2-depleted mice.^154^ Although these data argue for a pivotal role of ILC2s also in T cell replete mice during *N. brasiliensis* infection, anti-helminth immunity is based on a multilayer system involving several cell types including ILC2s and T cells.

Excessive type 2 immune responses can become detrimental and constitute the underlying mechanism in the pathogenesis of atopic disease including allergic asthma, atopic dermatitis, and allergic rhinitis. Increased ILC2 numbers were found in bronchoalveolar lavage (BAL) of asthmatic patients,^155^ in nasal polyps of patients with chronic rhinosinusitis^13^ and skin lesions of atopic dermatitis patients.^140,156^ In mouse models, multiple studies suggest a pivotal role for ILC2s in the pathogenesis of allergic asthma provoked by the inhalation of the protease papain, the fungal extract from *Alternaria alternata* or in airway hyperreactivity following influenza virus infection.^31,157,158^ Further, depletion of ILC2s in the papain model resulted in decreased Th2 cell numbers and type 2 inflammation. Moreover, depletion of ILC2s after the primary challenge with papain revealed defects in memory Th2 cell formation mediated by ILC2s and myeloid cells.^159,160^

ILC2 function was investigated in a mouse model of atopic dermatitis provoked by the vitamin D3 analog calcipotriol. While several studies have revealed that ILC2s promote atopic dermatitis, ILC2 activation in the skin might be in parts differentially regulated since ILC2s responded to IL-18, whereas the role of IL-33 in stimulating ILC2s appeared to be context dependent.^4,140,156,161^

Since the life cycle of helminths, such as *N. brasiliensis* creates tissue damage, e.g., the rupture of capillaries in the lungs, type 2 immune responses have not only developed to combat helminth infection but also to promote tissue repair and remodeling through secretion of molecules, such as AREG.^147,162^ AREG was secreted by ILC2s and acted as a growth factor that binds to the EGF receptors expressed on epithelial cells and promoted cell proliferation and tissue repair following influenza virus infection in the lung or during dextran sulfate sodium (DSS)-induced colitis.^163,164^

ILC2s contribute to acute inflammation in the context of hepatitis.^165^ However, ILC2s were found to mediate important functions in the resolution of chronic inflammation during arthritis by induction of regulatory T cells via ICOSL and GITRL.^143^ Moreover, multiple studies support a role for ILC2s in wound healing and tissue remodeling after infection or tissue damage in the skin, lung, and intestine.^147,163,164,166^ In addition, ILC2s also stimulated irreversible changes in the organ structure as they were implicated in liver fibrosis development.^167^

While limitations in specifically targeting ILC2s in the presence of T cells have to be considered when drawing conclusions from disease models, the experimental evidence for a role of ILC2s in promoting type 2 inflammation in the context of allergic asthma, atopic dermatitis, and helminth infections are encouraging and could provide novel targets for pharmacological intervention.

## ILC3s

### Formation of lymphoid organs and interaction in cryptopatches

**Fig. 5:**
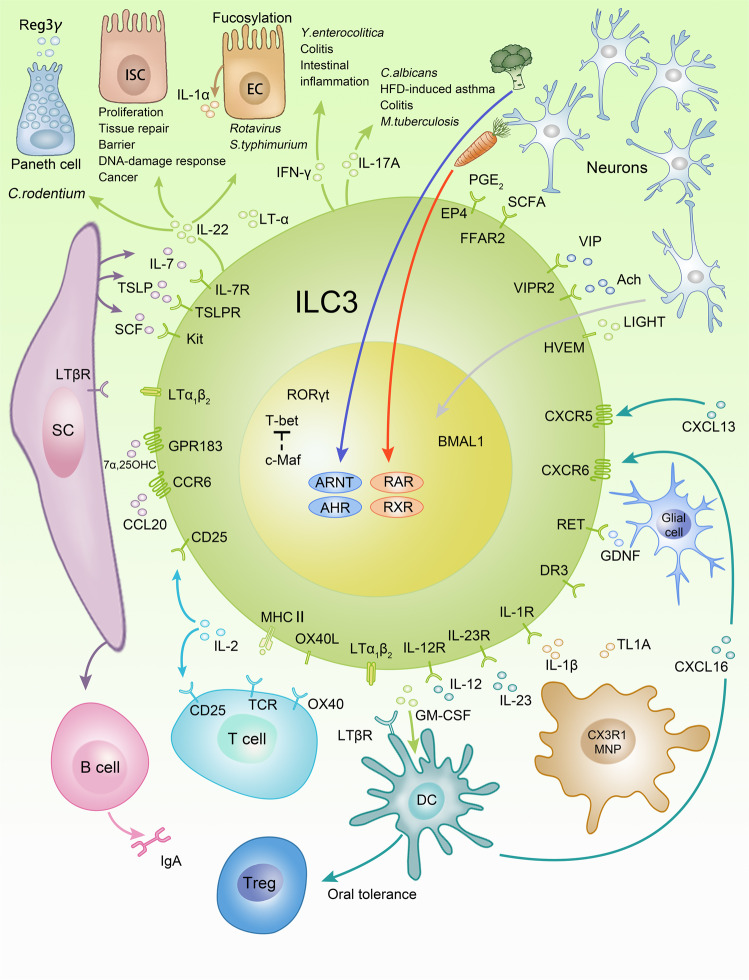
Immune regulatory functions of ILC3s. Regulatory circuits that involve ILC3s include interaction with myeloid cells, epithelial cells, stromal cells, neurons, and adaptive lymphocytes. Although depicted as one cell, it should be noted that CCR6^+^ ILC3s and CCR6^−^ ILC3s are developmentally and functionally distinct. MNP mononuclear phagocyte, 7α,25 OHC 7α25-hydroxycholesterol, M macrophage, SC stromal cell, EC epithelial cell, ISC intestinal stem cell, AHR aryl hydrocarbon receptor, Arnt nuclear translocator of AHR, RAR retinoic acid receptor, RXR retinoid X receptor, SCFA short chain fatty acid.

In the fetal period, LTi cells are essential for the formation of lymphoid organs, such as lymph nodes and Peyer’s patches. Following embryonic day 12.5, CXCR5^+^ LTi cells are clustering and form lymph node anlagen together with mesenchymal stromal cells. Formation of lymph node anlagen is promoted by retinoic acid and CXCL13 secretion thus attracting LTi cells. It was proposed that neuronal stimuli might trigger the initial steps in the formation of lymph node anlagen but this model is not yet supported by strong evidence.^168^ Factors promoting lymph organogenesis include IL-7, SCF, TSLP, TRANCE (RANKL), and LIGHT leading to the expression of LTα1β2 on ILC3s, which binds LTβ receptor on the mesenchymal cells, a decisive event in the formation of lymphoid organs (Fig. 5).^137,168^ The interaction results in further secretion of the chemokines CXCL13, CCL19, and CCL21, which attract adaptive lymphocytes to the lymph node anlagen and in the upregulation of adhesion molecules VCAM-1, ICAM-1, and Madcam-1, which facilitate the formation of lymphoid organs.^168^ This process is largely controlled by cell migration and the interaction of cell surface molecules during fetal development. Some of the molecular machinery is also maintained in CCR6^+^ ILC3s, the adult counterpart of LTi cells, which are located in cryptopatches in the intestine. CCR6^+^ ILC3s were attracted to the cryptopatches via the chemokine CCL20. Cryptopatch formation is altered in the absence of AHR or c-Kit-SCF signaling, which is controlled by AHR.^68,136^ Within cryptopatches, CCR6^+^ ILC3s interacted with myeloid cells via LTα1β2-LTβ receptor to stimulate secretion of IL-23, which resulted in increased IL-22 secretion and enhanced production of antibodies in cryptopatches.^169–171^ Cryptopatches were found in close proximity to nerve fibers, which were surrounded by glial cells. Glial cells secreted glial cell-derived neurotrophic factor (GDNF), which stimulated IL-22 production by ILC3s via the cell surface receptor Ret, and which provided protection in the context of *C. rodentium* infection and DSS-induced colitis.^172^

### Regulation of ILC3s

Similar to other ILCs, ILC3 activation is mainly regulated by soluble factors, such as cytokines, neuronal factors, metabolites, and inflammatory mediators. Important regulatory cytokines include IL-1α, IL-1β, IL-2, IL-7, IL-23, TL1A, SCF, and TSLP, which are secreted by a variety of cells including myeloid cells, T cells, epithelial cells, and stromal cells.^3^ ILC3s do not develop in the absence of IL-7, which is important for the development of most innate and adaptive lymphocytes. ILC3s produce IL-2 but also compete with T cells for this cytokine.^173,174^ IL-1β, IL-23, and TL1A are mainly produced by mononuclear phagocytes (MNPs), which are closely entangled with ILC3s, and which are potent stimulators of IL-22 production and ILC3 activation.^175,176^ TL1A induces expression of OX40L on ILC3s thereby promotes co-stimulation of T cells  during colitis.^177^ Epithelial IL-1α was shown to promote IL-22 production by ILC3s following rotavirus infection.^178^

While the crucial role for retinoic acid in formation of lymph organs was already discussed in the paragraph above, retinoic acid is also involved in the regulation of gut homing receptors by inducing a switch from CCR7 to CCR9 in ILC3s and ILC1s in gut-associated lymphoid tissue.^179^ Other molecules determining ILC3 migration include CXCR6 and G-coupled protein receptor GPR183, which sense CXCL16 and 7α25-hydroxycholesterol, respectively.^180–182^ As opposed to ILC2s, deficiency in retinoic acid causes diminished IL-22 production of ILC3s and susceptibility to *C. rodentium* infections.^135^ Similar to the role of AHR ligands in regulating the formation of postnatal lymphoid tissues, AHR ligands are crucial for IL-22 production and *Ahr*^−/−^ mice succumbed to *C. rodentium* infection.^67^ Further, ILC3s were stimulated by short chain fatty acid, via the FFAR2 receptor, which promoted IL-22 production and protection from colitis.^183^ Another study also identified prostaglandin E_2_ as an inflammatory mediator to prevent the systemic spread of bacteria by promoting barrier functions via the EP4 receptor on ILC3s and enhanced IL-22 production.^184^

In summary, ILC3 activation is regulated to a large extent by humoral factors released by myeloid cells and parenchymal cells in the tissue.

### Effector functions of ILC3s

IL-22 is the main effector cytokine secreted by both CCR6^+^ ILC3s and CCR6^−^ ILC3s. Although CCR6^−^ ILC3s produced less IL-22 than CCR6^+^ ILC3s, they remain to be an important source of IL-22.^21^ Unlike most interleukin receptors, the IL-22 receptor is mainly expressed on non-hematopoietic cells, especially epithelial cells.^185^ IL-22 maintained barrier integrity and the containment of intestinal bacteria at steady state and was shown to be protective in various infection models, including *C. rodentium*, *C. difficile*, *Rotavirus*, and *Salmonella* Typhimurium (*S. Typhimurium*) infection.^16,113,178,186,187^ *C. rodentium* infection is a model for attaching-and-effacing *Escherichia coli* (*E. coli*), which is controlled by IL-22.^16^ While mice deficient in IL-22 signaling were more susceptible to *C. rodentium* infection, the contribution of ILC3-derived IL-22 to the phenotype is still debated because CD4^+^ T cells, γδ T cells, and NKT cells are also potential sources of IL-22 in this model. ILC3s were found to be the major source of IL-22 in the first week of *C. rodentium* infection^20,187^ and were stimulated via IL-23 derived from Notch-2-dependent DCs or CX3CR1^+^ MNPs.^176,188^ Further studies suggest that CCR6^+^ CD4^+^ ILC3s are the main producers of IL-22, because depletion using an anti-CD4 antibody resulted in susceptibility to *C. rodentium* infection, whereas genetic depletion of NKp46^+^ ILC3s did not affect *C. rodentium* control on a T cell replete background.^187,189,190^ Altogether the studies argue for a role of T cells and ILC3s in controlling *C. rodentium* infection, however, a genetic model that selectively depletes all ILC3s without affecting other cell types is lacking.

IL-22 mediates downstream effects on different types of epithelial cells, such as Paneth cells, intestinal stem cells, and enterocytes via engagement of IL-22Rα1 and IL-10Rβ2 chains triggering a signaling cascade resulting in phosphorylation of STAT3.^191^ Activation of intracellular signaling resulted in secretion of antimicrobial peptides, such as Reg3β, Reg3γ, S108a, and S109a from Paneth cells. Furthermore, IL-22 promoted barrier functions in enterocytes, which limited the dissemination of bacteria. Together with LTα, IL-22 stimulated the glycosylation of epithelial cells that is crucial for protection against *S. Typhimurium*^186^ infection and IL-22 and interferon λ jointly stimulated IL-1α release during Rotavirus infection.^16,178,191^ IL-22 production protected intestinal stem cells from damage caused by chemotherapy, irradiation or genotoxic stress by metabolites, such as toxic AHR ligands, which could in turn modulate IL-22 production.^192–194^

Both tumor-protective and tumor-promoting effects of IL-22 were reported. IL-22 reduced inflammation and intestinal cancer in the acute phase of DSS-induced colitis by fortifying the epithelial barrier function and limiting the microbial influx. In the recovery phase, IL-22 was found to regulate epithelial proliferation.^17,192,195^ IL-22 limited the progression from epithelial damage to cancer transformation during chronic inflammation via induction of the DNA damage response in intestinal stem cells in a colorectal cancer model induced by DSS and azoxymethane.^192^ Tumor-promoting effects of IL-22 were found in adenomatous polyposis coli (APC)^Min/+^ mice, which develop colorectal cancer due to a mutation in the tumor suppressor gene APC.^195^ In this model, IL-22 promoted proliferation of epithelial cells and, therefore the availability of bioactive IL-22 controls cancer progression. Hence, mice deficient in IL-22-binding protein, which serves as a decoy receptor for IL-22, had more tumors, whereas *Il22*^−/−^ mice developed fewer tumors.^195^

Patients with mutations in *RORC* had selective immunodeficiency to *Candida albicans* (*C. albicans)* and *Mycobacterium tuberculosis* infections due to a reduction in IL-22 and IL-17A.^196^ While different cell populations that express *RORC* probably contribute to this drastic phenotype, ILC3s and especially IL-17A^+^ ILC3s, which were lacking in these patients, might be of relevance.^197^ IL-17A is mainly produced by CCR6^+^ ILC3s, consistent with T-bet as a negative regulator of IL-17A.^21^ While ILC3s were described as pivotal in the control of oral *C. albicans* infection, they could also trigger detrimental immune responses.^198^ These experiments were mainly carried out on a  *Rag*^−/−^ background, where ILC3s might be overactivated due to the lack of adaptive lymphocytes. In *Tbx21*^−/−^ (T-bet) *Rag*^−/−^ mice, IL-17A^+ ^ILC3s were shown to drive *Helicobacter typhlonius* triggered colitis.^69^ IL-17A derived from CCR6^+^ ILC3s was identified to be important for airway hyperactivity in the context of high-fat diet (HFD)-induced asthma, in which ILC3s were stimulated by IL-1β released from MNP in a Nlrp3 inflammasome-dependent manner.^199^

T-bet upregulation in CCR6^−^ ILC3s endows these cells with the functional properties of ILC1s including the production of IFN-γ. IFN-γ derived from ILC3s was shown to mediate both protective immunity against *Yersinia enterocolitica* (*Y. enterocolitica*) and *Clostridium difficile* (*C. difficile*) infection but could also trigger detrimental immune responses.^113,200^ Interestingly, during *Y. enterocolitica* infection ILC3s were activated via herpesvirus entry mediator (HVEM), a member of TNF receptor superfamily and soluble LIGHT.^200^ However, these cells were also shown to be activated by IL-12 and IL-23 and promote colitis in different models of intestinal inflammation including anti-CD40-induced colitis, *Helicobacter Hepaticus*-induced colitis, as well as immunopathology in the caecum induced by *S. Typhimurium* and *C. rodentium*.^21,24,189,190,201^

ILC3s modulate myeloid cell activation via secretion of GM-CSF and in this way indirectly regulate adaptive immune responses. Additionally, upon activation of IL-1β via MNP,  ILC3s are a crucial source of GM-CSF which promotes Treg cells and in this way mediates oral tolerance in the intestine.^202^ Moreover, ILC3-derived GM-CSF was shown to attract neutrophils to promote antibody production by marginal zone B cells.^203^

## Regulation of adaptive immune responses by ILCs

Unlike MHC I expression, which is present on almost every cell, expression of MHC II is considered as a hallmark of professional antigen-presenting cells. In parallel to antigen-presenting cells, ILC2s and ILC3s are also equipped with the machinery to process and present peptides on MHC II molecules and are therefore able to directly interact with T cells via MHC II-peptide-TCR complex.^154,204^ Expression of co-stimulatory molecules on ILCs, such as ICOSL, GITRL, and PD-1L was also reported to provide a second signal for T cell activation. Furthermore, IL-2 and GM-CSF secreted by ILC3s, in a myeloid-derived IL-1β-dependent manner, were found to be important for Treg cell-mediated tolerance induction.^174,202^ ILCs and T cells both express the high-affinity IL-2 receptor alpha-chain, CD25, which brings them in the position to compete for this essential growth factor, thus adding an additional layer of complexity to the regulation of T cell by ILCs.^173^

At steady state, CCR6^+^ ILC3s in the intestine present peptides on MHC II without providing co-stimulation, thus resulting in clonal deletion of the antigen-specific T cells, coined intestinal selection of T cells. Mice with deletion of MHC II in ILC3 developed intestinal inflammation, arguing that the intestinal selection of T cells is necessary to maintain immune homeostasis at mucosal sites.^173,204^ Further studies discovered that ILC3s upregulated co-stimulatory molecules such as OX40L after stimulation with TL1A or IL-1β during inflammation and thus promoted colitis via activation of pathogenic T cells.^177,205^

CCR6^+^ ILC3s are mainly positioned in cryptopatches in the adult intestine, which recruit B cells into the structure and thereby further mature. They are then referred to as isolated lymphoid follicles (ILFs) or mature ILFs.^19^ ILFs provide a structure for ILC3-B cell interaction and thus lead to a T-cell-independent stimulation of IgA, which has also been observed near the marginal zone in the human spleen.^171,203^ While IgA production in cryptopatches is considered to be T-cell-independent, it was reported that CCR6^+^ ILC3s modulate the interaction of follicular T helper cells and B cells via MHC II in the colon-draining lymph node and thus regulate the production of T-cell-dependent high-affinity IgA.^206^ Further, it was discovered that ILC3s support T-cell-dependent IgA via soluble LTα3 and T-cell-independent IgA via surface LTα1β2.^170^ Therefore, ILC3s can regulate Ig production via direct and indirect mechanisms.

Consistent with the expression of co-stimulatory molecules on their surface, ILC2s stimulate T cells via MHC II-TCR interaction. Stimulation of Th2 cells by ILC2s promoted IL-2 secretion by Th2 cells, which in turn activates ILC2s to produce IL-13 and promote resistance against worm infection.^154^ Interestingly, ILC2s could co-stimulate Th2 cells via PD-L1-PD-1 interaction and promote type 2 immunity.^207^ Moreover, IL-9-dependent expansion of ILC2s was found to be important for the induction of regulatory T cells via ICOSL and GITRL and resolution of chronic inflammation during arthritis.^143^ Taken together, these studies identify ILCs as pivotal regulators of adaptive lymphocytes that can either promote or dampen the adaptive immune responses depending on the environmental context.

## Regulation of ILC responses by the nervous system and by the circadian rhythm

The nervous system and the immune system are composed of complex sensory and effector structures, which continuously monitor homeostasis in tissues by measuring different parameters. Chemo-, mechano-, noci-, thermo-, and photo sensation prevail in the nervous system, whereas the immune system relies on non-self and self-recognition and to a lesser extent chemosensation.^208,209^ The effector molecules of the nervous system include neuropeptides and neurotransmitters, which transmit information over a short distance. In contrast, immune responses are mainly amplified by humoral factors, such as cytokines and direct cell-to-cell interaction. Functional specialization requires the constant exchange of information to efficiently coordinate responses between the immune and nervous system. In this context, recent research has revealed some of the pathways that define the crosstalk between ILCs and the nervous system.

The enteric nervous system is the largest accumulation of neurons outside the central nervous system and enteric neurons are closely entangled with ILCs.^208,209^ The crosstalk between neurons and the immune system is bidirectional since neurons express but also sense cytokines, such as TSLP, IL-4, and IL-31, which are known to boost type 2 inflammation.^210,211^ Furthermore, ILC2s produce neuropeptides, e.g., CGRP, which can also be released by neurons or pulmonary neuroendocrine cells (PNECs) and regulates lung inflammation and immunity against helminths. In addition to producing CGRP, ILC2s are also equipped with the receptor Ramp1/CALCRL to sense CGRP. CGRP triggered inhibitory signals in ILC2s and thus limited ILC2 activation in mouse models of helminth infection or allergic asthma.^212–215^ Additional neurotransmitters inhibiting ILC2 activation inculde the signature neurotransmitter of the sympathetic and parasympathetic nervous system norepinephrine and acetylcholine. These two neurotransmitters limit ILC2s via α7 nicotinic acetylcholine receptors and β2-adrenergic receptors^216,217^ and regulate allergic lung inflammation and worm resistance. Furthermore, acetylcholine signals from the vagal nerve stimulated ILC3s to secrete the immunoresolvent PCTR1 during peritoneal *E. coli* infections.^218^ In contrast, the neuropeptides neuromedin U (NMU) and vasoactive intestinal peptide (VIP) are expressed in the enteric nervous system and stimulate ILC2s via Nmur1 and Vipr2, respectively.^219–223^ NMU is expressed in cholinergic neurons in the intestine. NMU is upregulated following worm infection by excretory-secretory products of worms and regulates worm resistance via Nmur1.^221,222^ VIP is expressed by nociceptors in the lung and stimulates IL-5 production from several cell types including ILC2s, which act back on neurons to sustain lung inflammation via VIP.^220^ Similarly, VIP regulates IL-5 production from ILC2s and the number of eosinophils in the intestine and is dependent on circadian oscillations and nutrient intake.^219^

ILC2 and ILC3 activation in the intestine is controlled by the circadian rhythm, which allows to synchronize immunity and nutrient intake.^219,224–228^ Circadian regulation of ILC3s probably involves several mechanisms including the regulation of RORγt and Rev-Erbα by the Bmal1:Clock circuit, the regulation by the suprachiasmatic nuclei in the brain and release of the neuropeptide VIP, which is known to be controlled by circadian oscillations.^225–228^ Indeed, the deletion of Bmal1 in RORγt^+^ cells resulted in altered RORγt expression, diminished NKp46^+^ ILC3s and susceptibility to *C. rodentium* infection. Furthermore, VIP controlled IL-22 secretion by CCR6^+^ ILC3s via Vipr2. Seillet and colleagues found that Vipr2-deficient CCR6^+^ ILC3s produced less IL-22 and exerted reduced protective function in the DSS-induced colitis model.^225^ However, Talbot and colleagues reported that VIP inhibited CCR6^+^ ILC3s and conditional targeting of Vipr2 in ILC3s using *RORc*(γt)^Cre^ *Vipr2*^flox/flox^ mice resulted in an enhanced antimicrobial function of the intestinal epithelium against segmented filamentous bacteria but reduced lipid uptake.^224^ Since both stimulatory and inhibitory effects of VIP on CCR6^+^ ILC3s were observed, the function of this neuropeptide might be context-dependent or the discrepancy between these studies might be explained by differences in the experimental approach or the commensal microbiota. In total, these studies provide evidence that neuronal regulation of ILCs harmonizes immunity at barrier surfaces with the light-dark cycles, nutrient intake and homeostasis.

## Cytokine milieu and plasticity of ILC subsets

In parallel to T cell differentiation, where lineage-specifying TFs and downstream signaling mediators of the key cytokine receptors suppress disparate T cell fates, ILCs are negatively regulated by cytokine milieu or lineage-specifying TFs of other ILC subsets. In this context, IL-25 and TSLP were both reported to repress ILC3 activation and IL-22 production in the context of colitis and therefore exacerbate intestinal inflammation.^20,229^ Moreover, cytokines promoting type 1 immune responses, such as IFN-I, IFN-γ or IL-27 were found to inhibit ILC2s and consequently limit allergic inflammation.^31,132^ Similar findings were reported for the skin in the context of contact hypersensitivity, in which depletion of ILC2s resulted in an exaggerated type 1 immune response.^230^

Despite an established transcriptional program defined by a lineage-specifying TF, ILCs are able to adopt alternative cell fates after lineage commitment even at steady state but also in the context of chronic inflammation or a certain microenvironment. Plasticity is driven by down-modulation of the lineage-specifying TFs and induction of a different master TF that is often competing with the original TF in determining the cell fate. Plasticity of ILCs was originally observed in CCR6^−^ ILC3s, which lost expression of RORγt and upregulated T-bet, thus transforming ILC3s in a cell type that phenotypically mirrored ILC1s, such as expression of NKp46, NK1.1, NKG2D, IL12Rβ2, and IFN-γ. Since ex-RORγt^+^ ILC3s carry receptors for IL-12 and IL-23, they could drive chronic inflammation and immunopathology during colitis in patients with Crohn’s disease or in mouse models of experimental colitis induced by anti-CD40 treatment or infection.^21,24,25,189,190,201^ T-bet appears as a signaling hub that promoted the loss of RORγt in conjunction with the TF AIOLOS and by integrating diverse signals including Notch, IL-2, and IL-12 that support type 1 cell fate, whereas c-Maf and TGF-β suppress T-bet.^21,22,25,70,231^ Nevertheless, T-bet-deficient ILC3s were still able to promote detrimental immune response via the production of IL-17A during colitis in response to *Helicobacter typhlonius*.^69^ In humans, the differentiation of ILC3s into ILC1s could be reversed by the addition of IL-1β, IL-23 and retinoic acid in vitro.^232^

Differentiation towards an ILC1-like phenotype is not limited to ILC3s, but was also described for ILC2s and NK cells. Conversion of NK cells into ILC1-like phenotype was accompanied by down-modulation of Eomes and mediated by TGF-β, which is a signature cytokine acting on ILC1.^55^ ILC1-like NK cells were described in the tumor microenvironment or during cytomegalovirus or *T. gondii* infection. Notably, ILC1-like NK cells were distinct from both NK cells and ILC1s, were functionally impaired and failed to control tumor growth and cytomegalovirus infection.^233–235^ Plasticity of ILC2s towards ILC1 was described in the lungs of patients suffering from chronic obstructive pulmonary disease (COPD) and was promoted by the cytokines IL-1, IL-12, and IL-18 and connected to the induction of T-bet.^236^ ILC2 conversion to IL-17-producing ILC3s was reported in mice and humans. In mice, stimulation of ILC2s with IL-25 resulted in inflammatory ILC2s and conversion to IL-17 producing cells, which protect from *C. albicans* infection.^32,237^ This process was promoted by Notch signals, which induced upregulation of RORγt and IL-17A in ILC2s. Therefore, these cells were shown to be co-producers of IL-13 and IL-17A thus promoting allergic airway inflammation in the house dust mite model.^238^ In contrast, in humans, the conversion was promoted by ILC3-stimulating cytokines IL-1β, IL-23 and TGF-β in the context of psoriasis and nasal inflammation.^239^

ILC2s can switch their cytokine profile to IL-10 production.^240–242^ This process does not involve the upregulation of another lineage-specifying TF but is promoted by different cytokines including IL-2. Furthermore, it was discovered that IL-10 is under transcriptional control of the RUNX/CBF-β complex. Since IL-10 secretion by ILC2s is accompanied by loss of type 2 functional properties, it was also interpreted as exhaustion-like phenotype.^242^ While several groups accordingly reported IL-10 production by ILC2s under certain conditions,^240–242^ IL-10 was also proposed to constitute a separate regulatory ILC lineage defined by the transcriptional repressor ID3.^243^ However, since a recent paper published by Bando et al. failed to reproduce the existence of a separate ILC lineage different from known ILC populations, further research is needed to clarify the discrepancy between these findings.^241^

In summary, some degree of plasticity appears to emerge as a hallmark of ILC subsets and might constitute a mechanism of tissue adaption or immune regulation, however, more detailed in vivo fate mapping studies are required before definitive conclusions can be made regarding the generality of plasticity within ILC populations.

## Metabolic homeostasis

Low-grade chronic inflammation is determining energy expenditure in white adipose tissue (WAT), therefore, assigning an important role for the immune system in metabolic hemostasis. Among immune cells, ILCs, and predominantly ILC2s, are well represented in WAT. While type 2 inflammation in WAT is associated with increased energy expenditure, shifting the immune response towards type 1 immunity is associated with increased metabolic risks.^244^ ILC1s, which accumulated in WAT during obesity, were identified as an important source of IFN-γ. ILC1-derived IFN-γ was stimulated by IL-12 secreted by myeloid cells and supported the differentiation of M1 macrophages promoting obesity and insulin resistance.^103^

In contrast, ILC2s, whose numbers were decreased during obesity, were demonstrated to promote energy expenditure by diverse mechanisms including recruitment of AAMs.^148,245,246^ AAM differentiation is promoted by IL-4 mainly secreted by eosinophils but also NKT cells. ILC2s are recruiting eosinophils to WAT via secretion of IL-5.^148^ Besides the effect on AAMs, IL-4 can also directly act on adipocyte precursors that express the IL-4 receptor and promote differentiation of beige adipocytes.^246^ Beige adipocytes increase energy expenditure by uncoupling of the electrochemical gradient of the respiratory chain in the mitochondria.^244^ In addition to type 2 cytokine secretion, ILC2s promoted beiging of WAT by cleavage of the opioid peptide proenkephalin A (Penk) into bioactive methionine-enkephalin via prohormone convertase 1, an enzyme that has previously been associated with obesity.^244^ Penk stimulated beige adipocytes that expressed the opioid receptor δ1 resulting in upregulation of the uncoupling protein UCP-1, an increase in energy expenditure and a decrease in adipose tissue.^245^ ILC2 activation is regulated by IL-33 produced from adipocyte progenitors and mesenchymal stromal cells in WAT^124,125^ and controls UCP-1 in beige adipocytes via activation of type 2 inflammation.^247^ IL-33 also stimulated ILC2s in pancreatic islets, which then activated DCs to release retinoic acid and promote insulin secretion.^248^ Furthermore, metabolic homeostasis was linked to IL-22, which can be produced by ILC3s. IL-22 promotes metabolic homeostasis partially via regulating lipid absorption in the intestine, as well as additional mechanisms that require further investigation.^224,249^

## Future perspectives

The last 10 years of ILC research has identified previously unappreciated functional diversity of ILCs. ILCs have been detected in almost every tissue with enrichment at barrier surfaces. ILCs provide not only a first-line defense against infections, but they also have established many local signaling circuits to maintain broad tissue homeostatic functions. While some key signaling pathways mediating tissue homeostasis arise, these need to be further dissected, and additional circuits will likely be revealed. An emerging topic is the functional specialization of non-hematopoietic cells in regulating ILCs. Discovering the pathways utilized by tissue-resident cells to regulate ILC responses and tissue homeostasis has the potential to open up avenues for future research. To investigate these pathways will require new genetic tools to selectively and temporally interfere with parenchymal cells and ILC populations in diverse tissue microenvironments. Notwithstanding that, since ILCs have a similar molecular profile to T cells, one of the major unresolved limitations is still the specific targeting of ILC subsets without affecting adaptive lymphocytes. Given the overlap in effector molecules with T cells, the extent, or lack thereof, of functional redundancy between T cells and ILCs is an ongoing discussion.^250^ Moreover, future research will likely focus on pivotal immunoregulatory pathways controlled by ILCs that have the potential to be therapeutically harnessed in the context of health and disease.
